# Fifteen-Year Referral Trends and the Gender-Affirming Care Pathway in a Predominantly Adult Gender Dysphoria Cohort: A Retrospective Cohort Study from a University-Based Center in Türkiye

**DOI:** 10.3390/healthcare14193144

**Published:** 2026-09-22

**Authors:** Hanife Yılmaz Abaylı, Diğdem Göverti, İlay Dalkıran, Seray Sultan Karakoç, Aila Gareayaghi, Ezgi Şişman, Niyazi Umut Yıldırımoğlu, Aslıhan Polat

**Affiliations:** 1 Department of Psychiatry, Eyüpsultan State Hospital, 34075 Istanbul, Türkiye; 2 Department of Psychiatry, Faculty of Medicine, Kocaeli University, 41001 Kocaeli, Türkiye; digdem.goverti@gmail.com (D.G.); aila.gareayaghi@kocaeli.edu.tr (A.G.); umut.yildirimoglu@kocaeli.edu.tr (N.U.Y.); aslihan.polat@kocaeli.edu.tr (A.P.); 3 Department of Psychiatry, Dr. Yaşar Eryılmaz Doğubeyazıt State Hospital, 04400 Agri, Türkiye; ilay.dalkiran@gmail.com; 4 Department of Psychiatry, Balıklıgöl State Hospital, 63200 Sanliurfa, Türkiye; s.seraykarakoc@gmail.com; 5 Department of Psychiatry, Kocaeli City Hospital, 41060 Kocaeli, Türkiye; drezgisisman@gmail.com

**Keywords:** gender dysphoria, gender-affirming care, care pathway, presentation trends, transgender health, Türkiye

## Abstract

**Highlights:**

**What are the main findings?**

- Adult referrals increased overall across 15 years, an increase driven largely by the early years and flattening after 2017, while age at presentation and the predominance of trans men remained stable.
- Living outside the clinic’s province was associated with lower odds of reaching surgical evaluation, though this association did not persist in time-to-event analysis.

**What are the implications of the main findings?**

- Findings from Western adolescent-focused cohorts may not directly generalize to adult gender-affirming care in different sociocultural and legal settings.
- Residence relative to the center was associated with progression through the gender-affirming care pathway in exploratory analysis and may warrant attention when planning equitable service delivery.

**Abstract:**

Background/Objectives: Most long-term cohort data on gender-affirming care come from adolescent and young adult populations in Western Europe and North America. In a Muslim-majority setting where legal gender recognition is contingent on a court ruling, presentation trends and the care pathway of an adult-service gender dysphoria cohort remain unknown. This single-center, retrospective study describes 15-year presentation trends and the gender-affirming care pathway at a university-based center in Türkiye. Methods: Clinical records of 199 individuals presenting to a university gender identity clinic (2010–2024) were retrospectively reviewed, under routine institutional research consent obtained in writing from patients at intake; nine presented before age 18, and an adult-only sensitivity analysis was performed. Annual presentation counts were modeled with negative-binomial regression (accounting for overdispersion), with separate sub-period slopes estimated from 2015 and from 2017. Care-pathway stages (hormone board, surgical board, legal name change) were defined by board decision dates and cross-tabulated to show their overlap. Time-to-stage was analyzed with Kaplan–Meier estimation (with numbers at risk); predictors of reaching the hormone and surgical boards were assessed with Cox proportional-hazards models, censoring at the database closing date (25 November 2024). Results: The cohort comprised 67.3% trans men (AFAB) and 32.7% trans women (AMAB), approximately 2:1. Median age at presentation was 23.3 years (IQR 20.0–27.6). Annual presentations increased overall (negative-binomial IRR 1.21/year, 95% CI 1.06–1.37, *p* = 0.003), but the slope weakened from 2015 (IRR 1.07, 95% CI 0.86–1.34) and was flat from 2017 (IRR 0.96, 95% CI 0.70–1.31); neither age at presentation nor the proportion of trans men changed over time. Overall, 51% reached the hormone board, 41% the surgical board, and 31% a legal name change; stages were not strictly nested (12 individuals obtained a legal name change without surgical-board evaluation). In Cox models, endocrine pathology was associated with faster progression to the hormone board but not the surgical board; residence outside the center’s province was associated with lower odds of surgical-board attainment in logistic but not in time-to-event analysis. No prespecified between-group differences by sex assigned at birth were detected. Conclusions: This predominantly adult Turkish cohort shows an overall long-term increase in referrals—concentrated in the earlier years—with age at presentation and the recorded AFAB/AMAB distribution statistically stable across 15 years. Residence relative to the center was associated with surgical-board attainment in exploratory analysis only.

## 1. Introduction

Gender-affirming care is a multidisciplinary model encompassing the medical, psychological, and social services that support the identities of transgender and gender-diverse individuals. This model is guided by current international standards such as the World Professional Association for Transgender Health’s Standards of Care, Version 8 (WPATH SOC-8) [1]. It also draws on the European Network for the Investigation of Gender Incongruence (ENIGI) protocol [2]. SOC-8 emphasizes that psychiatric assessment should be supportive rather than serving as a mandatory gatekeeping mechanism [1]. This approach forms part of the broader international shift toward the depathologization of gender diversity reflected in ICD-11 [3]. The global health burden and unmet needs of transgender individuals are increasingly well documented [4], and prevalence estimates continue to rise [5]. The clinic whose records we analyze operates within this gender-affirming, person-centered framework, in which psychosocial assessment is intended to be supportive rather than gatekeeping. A note on terminology: we use “gender-affirming” and “trans men/trans women” throughout; the term “gender dysphoria” is retained only where it denotes the formal diagnostic category in force during the study period or the name of a clinical service, and is not used as a descriptor of transgender identity itself. 

Over the past fifteen years, cohort studies from gender clinics in high-income countries have reported two consistent trends: a marked increase in the number of referrals, and a decrease in age at presentation. This has been accompanied by a rising proportion of individuals assigned female at birth (AFAB)—a pattern often described as “youthification”. However, the vast majority of this literature has been derived from adolescent and young adult populations in Western Europe and North America. Recent cohort studies from Sweden [6], Norway [7,8], Austria [9], Denmark [10,11], France [12], Colombia [13], Germany [14], the United Kingdom [15], South Korea [16], and Finland [17] are predominantly focused on pediatric/adolescent services. By contrast, adult-focused data from non-Western settings—such as Iran [18,19], India [20,21], and Taiwan [22]—remain comparatively scarce. Most such studies are single-axis in focus, addressing either epidemiology or surgical experience alone, and research output on this topic from Muslim-majority countries is likewise limited [23]. Moreover, “Western” health systems are themselves far from homogeneous—access pathways and clinical procedures differ substantially between, for example, Italy, Belgium, Denmark, and the United Kingdom—so comparisons should avoid treating them as a single group. Whether these trends generalize to adult populations across differing legal, cultural, and health-system contexts therefore remains largely unknown. 

The mental health burden borne by transgender individuals is largely explained through the framework of minority stress arising from stigma, discrimination, and social exclusion [24]. This framework has been specifically adapted for transgender and gender-diverse people, whose stressors include gender-related victimization, non-affirmation, internalized transphobia, and the concealment of identity [25,26]. This burden manifests as elevated rates of conditions such as depression and anxiety among transgender individuals [27] and is compounded by stigmatizing interactions within healthcare settings [28], and it is increasingly understood through an intersectional lens, in which gender-minority status compounds with other social and structural disadvantages [29,30]. It becomes even more pronounced in contexts marked by substantial legal and sociocultural barriers [31,32,33]. For transgender people in Türkiye, these general barriers are compounded by documented discrimination in employment, housing, and healthcare, limited legal protection against discrimination, and concentration of specialized services in a few urban centers, all of which can delay or obstruct access to care. Türkiye offers a distinctive context in which to examine this gap. As a country in which secularism coexists with a Muslim-majority social structure, legal gender transition has been regulated since 1988; however, the current Turkish Civil Code (No. 4721, Article 40) requires a court ruling in addition to a medical board report. Until 2017, legal recognition was contingent upon a mandatory sterilization requirement, which was abolished that year. This dual (medical + judicial) structure, combined with the psychiatric evaluation and real-life-experience requirements that have applied in this clinic’s pathway during the study period [34], produces a care pathway distinct from that of many Western systems. 

Within this framework, the Kocaeli University Gender Identity Clinic (KoUGIC), established in 2004, is one of Türkiye’s pioneering multidisciplinary centers and functions as a national referral center. The clinic’s care model, multidisciplinary board structure, and sociopolitical context have recently been described in detail; however, that descriptive account did not present systematic outcome data and explicitly deferred quantitative findings to future dedicated studies. The present study aims to fill precisely that gap.

The objectives of this study are: (1) to characterize temporal trends in the number of referrals, age at presentation, and the proportion assigned female at birth between 2010 and 2024; (2) to quantitatively map the care trajectory from initial presentation to the hormone board, the surgical board, and legal name change; and (3) to identify predictors of reaching these milestones, with particular attention to inequities in geographic access.

## 2. Materials and Methods

### 2.1. Study Design and Sample

This is a single-center, retrospective cohort study, reported in accordance with the STROBE (Strengthening the Reporting of Observational Studies in Epidemiology) guidelines [35]. Records of all individuals who presented to the Kocaeli University Gender Identity Clinic between 2010 and 2024 and underwent clinical evaluation were reviewed. Although the clinic provides adult care, first-contact records include a small number of late-adolescent individuals (aged < 18 at presentation: *n* = 9, 4.5%); these individuals were retained because they transitioned into adult follow-up. The cohort comprised all individuals evaluated for gender dysphoria regardless of eventual transition goal; gender identity was recorded on a binary trans man/trans woman axis in the clinical file, and a distinct non-binary identity category was not separately coded, so non-binary individuals—if present—could not be identified as such in these records. 

Records were assembled from the clinic’s data sources: a primary case spreadsheet, two supplementary sheets, and the multidisciplinary board and legal-procedure records held in the clinical file. Across all sources, 215 records were identified. Records were linked and de-duplicated on the clinic file number; one exact duplicate and one record with a blank file identifier were removed, yielding 213 unique individuals. Inclusion in the descriptive cohort required a core clinical record with a recorded sex assigned at birth; on this basis 199 individuals formed the main cohort (Figure 1). Two of these 199 had a presentation date that could not be validated; they were retained in the descriptive cohort but excluded from all date-dependent analyses, as described below. Date-dependent analyses (annual trend and time-to-event models) were therefore restricted to the 197 individuals with a validated presentation date. Fourteen individuals whose records lacked the recorded sex field (8/14) or appeared only in the supplementary sheets were set aside from the primary analysis and examined separately in a sensitivity analysis (*N* = 213). One index presentation per person was used; repeat presentations were not separately modeled. Chart abstraction was performed from the existing de-identified records by the clinical authors using the fixed record fields listed above; a formal inter-rater reliability assessment was not performed because the principal outcomes were abstracted from predefined, objective record fields; nevertheless, residual abstraction error cannot be excluded, and this is noted as a limitation. Missing data for each key variable and outcome are reported in Table 1 and the Results; all models were complete-case, and the analytic sample is stated for each model. 

### 2.2. Variables

Sociodemographic variables included sex assigned at birth (recorded as a binary field, female or male, as entered in the clinical record; intersex status was not separately elicited and no intersex individuals were identified in the records), transition direction—reflecting sex assigned at birth relative to affirmed gender (trans men [assigned female at birth; AFAB], trans women [assigned male at birth; AMAB]), date and age at presentation, and province of residence. Age at presentation was calculated from the dates of presentation and birth. Stages of the care journey were defined on the basis of board dates recorded in the clinical file—the multidisciplinary board approval that gates each step under the Turkish legal framework. Board dates were used in preference to hormone-initiation or procedure dates because they were the most completely and reliably documented milestones in the record, and because in this legal context board approval is the formal decision point that determines whether a patient may proceed. The stages were: (i) referral to the hormone (Gender Affirmation) board, (ii) referral to the surgical board, and (iii) legal name change. Individual surgical-procedure fields, by contrast, contained a high proportion of missing data (10–40% “unknown”) and were therefore used only as a supplementary measure rather than as a primary outcome. Endocrine comorbidity was derived as a binary variable (any pathology present/absent) from the endocrinology consultation record. For the MMPI, rather than standardized scale T-scores, we used the holistic interpretations of clinicians experienced in gender dysphoria care. This was a deliberate choice: several MMPI items and scales embed binary sex/gender-based assumptions, and its normative samples do not include trans people, so raw scale elevations can be misleading in this population. The clinicians’ original free-text interpretations were retrospectively grouped by the research team into four categories (within normal limits/elevated clinical or personality-scale features/defensive response set/invalid or non-interpretable profile); this recategorization was performed by consensus against the recorded interpretive text and did not re-score the raw MMPI data. The MMPI was one input to a broader clinical assessment that included diagnostic interviews; it was not the sole basis for any diagnosis. The standardization limitation of this approach is addressed further in the Discussion and Supplementary Material. The geographic tiers were classified at province level: residence in Kocaeli (the center’s province; in-province), a neighboring province (sharing a land border with Kocaeli), or a distant province (all others); this is a province-level ecological proxy and not a measure of actual travel distance or time. Legal name change was ascertained from the legal-procedure record in the clinical file; because a name change completed outside the center may not be captured, this outcome may be under-ascertained, and it does not necessarily follow surgical-board assessment. Endocrine pathology denotes any documented endocrinological pathology (e.g., polycystic ovary syndrome, thyroid or other endocrine disorder) recorded at the endocrinology consultation, coded as present/absent. Age at diagnosis and group-therapy attendance, where reported in tables, are descriptive record fields and were not primary outcomes. 

### 2.3. Statistical Analysis

Our analytic strategy was organized around the study’s three objectives—describing temporal trends, mapping the care pathway, and identifying predictors of pathway progression—and each test was chosen to match the data type of the corresponding outcome (count, time-to-event, or binary) rather than to apply a single uniform model. Temporal trends were assessed primarily with regression across calendar year (the continuous, more parsimonious approach); the pre- versus post-2017 dichotomy is reported only as a descriptive companion because 2017 marks the abolition of the sterilization requirement, a concrete legal change in substantive interest, and is not used to make causal claims or as the primary trend model. Annual presentation counts were modeled with regression on calendar year. Because the counts were overdispersed (Pearson χ^2^/df = 4.5), negative-binomial regression was used in preference to Poisson (incidence rate ratio, IRR). Rather than a single piecewise model with estimated breakpoints, we fitted separate negative-binomial regressions restricted to the sub-periods from 2015 onward and from 2017 onward and report each sub-period slope with its 95% confidence interval; we describe these as separate sub-period analyses, not as a segmented-regression model. The pre/post-2017 comparison (2010–2016 vs. 2017–2024) is descriptive only and is not interpreted as an effect of the 2017 legal reform, as no interrupted-time-series model was fitted and concurrent changes cannot be excluded. The trend in age at presentation was assessed with linear regression and Spearman correlation, and the trend in the proportion of trans men with logistic regression. Time to reaching the hormone and surgical boards was estimated from the date of presentation using the Kaplan–Meier method, with numbers at risk reported. Individuals who had not reached a given board were administratively censored at the database closing date (25 November 2024, the latest recorded date in the dataset); the same fixed closing date was applied to all individuals, because board and clinical records were reviewed uniformly up to that date. We assume non-informative censoring, acknowledged as a limitation because patients who stopped attending or transferred care cannot be distinguished from those still in follow-up. Fourteen individuals (seven for each board) were known to have reached a board but had no recorded decision date; because these are events with an unknown event time rather than censored non-events, they were excluded from the corresponding time-to-event analysis rather than treated as censored, and a sensitivity analysis in which these undated events were re-included with their event time imputed at the board-specific median observed event time confirmed that their exclusion did not materially change the estimates (hormone-board geographic HR 0.65 vs 0.70; surgical-board 0.64 vs 0.70). Date-dependent analyses were restricted to the 197 individuals with a validated presentation date (two of the 199 descriptive-cohort members lacked one and were excluded from all date-dependent analyses, including the annual trend, which is why Supplementary Table S3 sums to 197). From these 197, the time-to-event analytic sample for each board was derived separately, with non-overlapping exclusions. For the hormone board: seven individuals who reached the board but had no recorded decision date (events with unknown timing, not censored) and two whose recorded decision date preceded the presentation date (a data-entry inconsistency yielding a negative interval) were excluded, giving 188 individuals and 93 events. For the surgical board: the seven undated events were excluded and no negative-interval events occurred, giving 190 individuals and 74 events. The two negative-interval hormone events involved individuals who remained valid surgical-board events, so they are retained in the surgical-board analysis. Because the crude medians among those reaching a board differ from Kaplan–Meier medians, both are reported and clearly distinguished. Legal name change was not modeled with survival methods because it is court-administered, is not downstream of board evaluation, and had incomplete dating. Predictors of reaching the hormone and surgical boards were analyzed with Cox proportional-hazards models (time origin: presentation; event: board decision; censoring as above). The proportional-hazards assumption was tested with Schoenfeld residuals; age at presentation and sex assigned at birth showed time-varying effects in both models and were handled by stratification (age in three groups: <20, 20–25, and >25 years), leaving residence relative to the center and endocrine pathology as covariates, both of which satisfied the assumption. The adjustment set was chosen a priori on clinical grounds. Endocrine pathology occurred in only 14 individuals, so its estimate was additionally checked with penalized Cox regression (lifelines CoxPHFitter, ridge penalizer 0.1) given the risk of sparse-data bias. Full logistic-regression results for the same outcomes are provided in the Supplementary Material (Table S2) for comparability with prior work; the Cox models are primary. Effect sizes for Table 1 were computed as the rank-biserial correlation for continuous variables (derived from the Mann–Whitney U statistic) and as the odds ratio (with a Haldane–Anscombe +0.5 continuity correction applied to all cells) for binary variables; the 95% confidence interval for the between-group median difference was obtained by percentile bootstrap (5000 resamples), and confidence intervals for odds ratios were the standard Wald intervals. Because Table 1 involves multiple between-group tests, we additionally applied a Benjamini–Hochberg false-discovery-rate correction and, more conservatively, a Bonferroni correction; no comparison was statistically significant before correction (all raw *p* > 0.05), so none became significant after either adjustment. We therefore interpret Table 1 descriptively and do not treat any single comparison as confirmatory. The significance threshold was two-sided *p* < 0.05; *p*-values below 0.001 are reported as *p* < 0.001. Analyses used Python 3.11 (pandas 2.2, statsmodels 0.14, lifelines 0.30, scipy 1.13). 

### 2.4. Ethics

Approval was obtained from the Kocaeli University Faculty of Medicine Non-Interventional Clinical Research Ethics Committee (decision no. KÜ GOKAEK-2025/08/17; project no. 2025/181; date 10 April 2025). The study was conducted retrospectively in accordance with the Declaration of Helsinki. All direct identifiers (name, contact information, and file number) were permanently removed before analysis and no linkage key linking study identifiers back to individuals was retained by the analysts; the working dataset was therefore anonymized rather than pseudonymized. Because the dataset concerns a small, potentially identifiable population and includes nine individuals who were younger than 18 at presentation, access was restricted to the clinical study team, data were stored on institutional password-protected systems, and no cell counts that could identify an individual are reported. The ethics committee approval covered the retrospective use of all records, including those of the nine individuals who were under 18 years at first presentation; for these individuals, the routine institutional research consent for use of de-identified clinical data was provided by a parent or legal guardian, together with the adolescent’s own assent. 

### 2.5. Researcher Positionality (Reflexivity)

A subset of the authors are clinicians practicing at the clinic under study. While this insider position enabled an in-depth interpretation of clinical processes and record structure, all outcome definitions were grounded in predetermined, objective record fields (board dates, legal procedure records). The analyses were conducted using standard statistical methods in order to limit potential interpretive bias. Because several authors are simultaneously clinicians in the service being evaluated, a dual-role and potential conflict of interest exist: the same team both delivers the care and reports on its outcomes. We mitigated this by defining all outcomes on objective, pre-existing record fields and by not making evaluative claims about the quality of the service’s own decisions. We also note transparently that, as a retrospective record review, the study did not incorporate direct transgender and gender-diverse involvement in its design, analysis, or interpretation, and to our knowledge the author team did not include openly transgender members; we therefore cannot claim that trans perspectives were represented in the research process, and we recommend community-based participatory approaches, and inclusion of trans researchers and advisors, for future work.

## 3. Results

### 3.1. Cohort Characteristics

Of the cohort, 67.3% (*n* = 134) were trans men (assigned female at birth; AFAB) and 32.7% (*n* = 65) were trans women (assigned male at birth; AMAB), a ratio of approximately 2:1. The median age at presentation was 23.3 years (IQR 20.0–27.6; range 16.6–53.4; aged < 18 at presentation: *n* = 9, 4.5%). Of the cohort, 66.3% resided in Kocaeli, and the remaining participants resided in 20 other provinces. No statistically significant differences were observed between sex-assigned-at-birth groups in age at presentation, age at diagnosis, place of residence, endocrine comorbidity, or progression through any stage of the care pathway (all *p* > 0.05). The corresponding effect estimates and their 95% confidence intervals, all of which included the null value, are presented in Table 1. 

### 3.2. Fifteen-Year Presentation Trends

Annual presentations increased overall between 2010 and 2024. Because the counts were overdispersed (Pearson χ^2^/df = 4.5), a negative-binomial model was used: incidence rate ratio (IRR) 1.21/year (95% CI 1.06–1.37, *p* = 0.003). This overall increase was driven largely by the earliest years; the slope weakened from 2015 (IRR 1.07, 95% CI 0.86–1.34, *p* = 0.54) and was flat from 2017 onward (IRR 0.96, 95% CI 0.70–1.31, *p* = 0.78). A descriptive comparison of mean annual counts (4.3/year in 2010–2016 vs. 20.9/year in 2017–2024, an approximately 5-fold difference) is presented for context only and is not interpreted as an effect of the 2017 legal reform. Annual counts are provided in a Supplementary Table S3 to allow reproduction of the trend analysis. Neither age at presentation (Spearman ρ near zero, *p* = 0.997) nor the proportion of trans men (logistic trend *p* = 0.674) changed significantly over time (Figure 2; Table 2). 

### 3.3. Gender-Affirming Care Pathway

Of the 199 individuals who presented, 102 (51%; 95% CI 44–58) reached the hormone board, 81 (41%; 95% CI 34–48) reached the surgical board, and 62 (31%; 95% CI 25–38) obtained a legal name change. The stages were not strictly nested: 12 individuals obtained a legal name change without a surgical-board evaluation (10 without reaching any board), and two reached the surgical board without a hormone-board record. A full cross-tabulation of stage combinations is given in Table 3. Because legal name change is not downstream of surgical evaluation, stage-specific proportions are reported against the full cohort (*N* = 199) rather than as sequential transition percentages; among those reaching the hormone board, 79/102 (77.5%) also reached the surgical board. The largest decrease occurred at the first stage, where approximately half of those presenting did not reach the hormone board (Figure 3; Table 3). Among those who reached a board, the crude median time from presentation was 0.95 years for the hormone board and 1.91 years for the surgical board; the corresponding Kaplan–Meier medians, which account for individuals who had not yet reached the board, were longer (2.77 and 6.27 years, respectively), reflecting the substantial proportion who never reached each stage within the observation window (time-to-event analyses: hormone board *n* = 188, 93 events; surgical board *n* = 190, 74 events). Median follow-up from presentation was 4.9 years (IQR 2.5–6.9; range 0.0–14.5; 1008 person-years). 

### 3.4. Predictors of Reaching Each Stage

In Cox proportional-hazards models (stratified by age group and sex assigned at birth, whose effects were time-varying; censored at the database closing date, 25 November 2024; hormone board 188 individuals, 93 events; surgical board 190, 74 events), endocrine pathology was associated with faster progression to the hormone board (HR 2.11, 95% CI 1.09–4.06, *p* = 0.026; penalized estimate HR 1.93, *p* = 0.042) but not the surgical board (HR 1.60, 95% CI 0.78–3.30, *p* = 0.201; penalized estimate HR 1.51, *p* = 0.24). Residence outside the center’s province, associated with lower odds of surgical-board attainment under logistic regression (OR 0.50, 95% CI 0.25–0.98, *p* = 0.045), was not significant in the time-to-event framework (hormone board HR 0.70, 95% CI 0.44–1.11, *p* = 0.128; surgical board HR 0.70, 95% CI 0.41–1.17, *p* = 0.174); we therefore treat the geographic association as exploratory (Figure 4; Table 4). Fourteen individuals (seven per board) reached a board on record but lacked a decision date and were excluded from the time-to-event models as events with unknown timing rather than censored; their inclusion as a sensitivity check did not materially change the estimates.

### 3.5. Geographic Access

The rate of reaching the surgical board was 45% in Kocaeli, 29% in neighboring provinces, and 39% in distant provinces. The ordinal distance tier did not show a significant monotonic gradient (OR 0.68/tier, *p* = 0.118); the difference was associated mainly with the binary distinction between residence within versus outside Kocaeli (Figure 5), which is more consistent with a province-level pattern than with a distance-dependent effect. Because this is a crude province-level proxy and the association did not persist in time-to-event analysis, it is reported as exploratory. 

### 3.6. Psychometric Profile (MMPI)—See Supplementary Material

Because MMPI testing was selective (143/199, 72%) and based on non-standardized clinician interpretation rather than standardized scale scores, the psychometric data are reported descriptively in the Supplementary Material (Table S1, Figure S1) rather than in the main analysis. Those assessed differed from those not assessed in the rate of reaching the surgical board (48% vs. 21%, *p* = 0.001), indicating that testing was not random; the profiles are therefore not interpreted as representative of the cohort. 

### 3.7. Sensitivity Analysis

When the 14 individuals initially set aside were included (*N* = 213), the core findings were essentially unchanged. Among these 14, eight lacked a recorded sex assigned at birth; the trans-men proportion of 65% in the *N* = 213 cohort is therefore computed over the 205 individuals with a recorded value (rather than all 213). The overall increase in presentations persisted (negative-binomial IRR 1.21/year, 95% CI 1.07–1.37, *p* = 0.003), and pathway-attainment rates shifted by no more than 3 percentage points (hormone board 49%, surgical board 39%, legal name change 30%). The exploratory geographic association was of similar direction. In an adult-only sensitivity analysis excluding the nine individuals younger than 18 at presentation and the two with a missing presentation age (analytic *n* = 188 with complete age data; 190 excluding only the under-18 group), all main findings were again essentially unchanged (negative-binomial trend IRR 1.23, 95% CI 1.06–1.42, *p* = 0.001; trans-men proportion 67.6%; pathway attainment within 1 percentage point of the full cohort). 

## 4. Discussion

Fifteen years of referral data from a single adult gender dysphoria clinic in a Muslim-majority setting, where legal transition requires a court ruling, allow us to test how well the international literature generalizes beyond the contexts in which it was generated. Our findings align with the literature on some dimensions and diverge from it on others.

Referral volume increased over the 15 years overall (negative-binomial IRR 1.21/year), an increase concentrated in the earlier years and flattening after 2017, with trans men outnumbering trans women roughly 2:1—consistent with high-income-country gender clinic reports over the past decade. Age at presentation is where our cohort parts ways with the literature. Unlike the “youthification” reported elsewhere, our sample’s median referral age held steady at approximately 23 across all 15 years, with no significant shift in sex ratio. Some of this reflects visibility: youthification—a declining referral age among adolescents (as described in the literature)—cannot register structurally in a clinic that predominantly serves adults. Thus, the stability we report should be read as stability in adult presentation age, not as evidence that youthification is not happening in Türkiye—we simply cannot see the population in which it would show up. Adolescent-focused cohorts in Sweden [6] and Norway [8] report both youthification and an accelerating sex-ratio shift; our data cannot speak to whether the same is true here. The legal age threshold for hormone therapy, the predominantly adult composition of our sample, and limited adolescent access to specialized services all push in the same direction—one misaligned with Western cohort data. Beyond these structural factors, the absence of a youthification signal and of a rising trans-men proportion may also reflect context-specific influences: lower social visibility and higher anticipated stigma for gender-diverse youth in a Muslim-majority setting, family-mediated help-seeking that can delay adult presentation, and the concentration of specialized services in a few centers, all of which may shape who reaches an adult clinic and when. These remain conjectural in the absence of adolescent-service and population-level data.

Approximately half of those presenting reached the hormone board—the largest decrease along the pathway, occurring at entry, where mandatory psychiatric evaluation, a real-life-experience requirement, multidisciplinary board sign-off, and a court process apply. WPATH SOC-8 specifies that psychiatric evaluation should support rather than gatekeep [1], whereas Türkiye’s legally mandated steps sit in tension with this standard. To our knowledge, this is among the first quantitative descriptions of that entry bottleneck in an adult-service setting. Time-to-stage should be read with the distinction between crude and Kaplan–Meier medians in mind: among those who reached a board, the crude median was 0.95 years to the hormone board and 1.91 years to the surgical board, whereas Kaplan–Meier medians accounting for censoring were longer (2.77 and 6.27 years, respectively). Although gender-affirming interventions have been associated with improved body image and quality of life in other studies [19,36], our data include no mental-health or quality-of-life outcomes, so we cannot demonstrate such benefits here or evaluate whether the pre-hormone waiting interval carries a cost; we raise this only as external context. Similar trajectory analyses exist for adolescents in Norway [8], Austria [9], and Denmark [10,11]; ours extends the approach to a different age group under a different legal regime. The field is concurrently addressing adjacent areas—hormone continuation and discontinuation [37], surgical approach [38,39], surgical-volume trends [40], fertility preservation [41], and body-composition changes during treatment [42]—and our data complement rather than replace that work. Two pathway findings warrant comment. First, individuals with a documented endocrine pathology reached the hormone board more often than those without (86% vs. 49%). Although a prior endocrine condition might intuitively be expected to complicate or delay hormone therapy, one possible explanation here is structural: these patients are already engaged with endocrinology and the hospital system, so the referral and board process is easier to navigate—an ascertainment/access effect rather than a clinical one. Second, the progressive decrease in numbers across pathway stages is substantial (roughly half do not reach the hormone board, and fewer still the surgical board). Plausible contributors—none directly measured here—include the mandatory evaluation and waiting requirements at entry, the indirect costs of repeated attendance, individual preferences (not every person seeks every intervention), completion of some steps outside the center, and ongoing follow-up in which a later stage has simply not yet been reached rather than been abandoned. Distinguishing genuine attrition from these alternatives will require prospective, patient-reported data.

The geographic association was the most notable exploratory finding: under logistic regression, patients living outside the center’s province were less likely to reach the surgical board than those living within it, though this association did not persist in the time-to-event models and should therefore be read cautiously. The pattern was not a monotonic distance gradient—patients from distant provinces fared no worse than those from neighboring ones—so physical distance alone is unlikely to be the mechanism. A plausible but unmeasured contributor is cumulative indirect cost (repeated board visits, pre- and post-surgical follow-up, travel, accommodation); because we did not measure travel distance or cost, socioeconomic status, referral pathways, or patient preferences, we present this only as a hypothesis for future research. The observed difference may equally reflect local referral processes, clinic familiarity, insurance, or unmeasured selection. A geographic disparity was flagged as a limitation in a previous descriptive report from this clinic [34]. We have no direct measures of mental health, discrimination, or quality of life, so minority stress functions here as an explanatory frame rather than a measured outcome; if the association is real, it would point to a structural, access-level factor alongside the interpersonal mechanisms emphasized in minority stress theory [24]. This pattern is usefully read through an intersectional lens [29]: gender-minority status does not act in isolation but can compound with geographic and structural disadvantage, so that living far from a specialized center may add to, rather than simply sit beside, the barriers a trans person already faces. We could not, however, resolve finer geographic contrasts—for example between large metropolitan provinces (such as İstanbul) and rural areas—because outside the center’s province the per-province counts were small (mostly single-digit), leaving the binary in-province versus out-of-province contrast as the only stable comparison. A larger multi-center sample would be needed to test whether the disadvantage is specifically rural, metropolitan-peripheral, or distance-related. 

Trans men and trans women showed similar sociodemographic profiles and care-pathway attainment, with no statistically significant differences detected. Given the modest sample, these non-significant tests should not be read as establishing equivalence between the groups. 

Detailed psychometric (MMPI) findings are presented as a descriptive supplement (Supplementary Material), given that testing was selective and based on non-standardized clinician interpretation rather than standardized scoring. More broadly, the international depathologization of gender diversity [3] and long-term evidence that regret after transition is uncommon [43], together with methodological cautions about non-probability sampling in minority-stress research [44], frame how such psychometric data should be interpreted. 

### Limitations

Several limitations temper how these results should be read. The single-center, retrospective design leaves the data susceptible to missing records and to whatever selection pressures are specific to this particular clinic. Data on individual surgical procedures were, in practice, too incomplete to serve as a primary outcome—a real gap, and one that pushed us toward board dates as the more reliably documented marker of the care trajectory. That substitution was a compromise, not a preference.

Sample size is a genuine constraint, especially for the trans-women subgroup (*n* = 65; AMAB), where statistical power to detect small effect sizes is limited. Readers should treat the absence of a between-group difference as “no significant difference was detected,” not as evidence that no difference exists—the study simply was not powered to rule one out. A related problem sits in the early years of the cohort: presentations between 2010 and 2016 were few enough that trend estimates for that period can shift with small fluctuations in the data.

The MMPI findings need a caveat of their own. Rather than relying on standardized clinical-scale T-scores, we used interpretations from clinicians experienced in gender dysphoria care. That choice was deliberate—raw MMPI scores carry limited validity in this population, given that the instrument’s normative samples are non-trans and built on binary sex assumptions. But because those interpretations were not standardized across raters, the MMPI-related results are descriptive at best and shouldn’t be over-read. Several specific limitations follow from this design. First, the four interpretive categories (within normal limits, elevated clinical or personality-scale features, defensive attitude, and invalid) were assigned by clinicians rather than derived from fixed cut-offs, so inter-rater reliability could not be quantified and some misclassification is likely. Second, the MMPI was administered selectively rather than to every patient (*n* = 143 of 199): testing was ordered at clinician discretion, typically when the assessing team judged that additional psychometric information would aid a complex evaluation, rather than by a fixed protocol. This non-random selection raises the possibility of indication bias—indeed, those tested reached the surgical board more often than those not tested (48% vs. 21%)—so the assessed subgroup is not representative. Importantly, the MMPI was only one component of a broader clinical assessment that included diagnostic interviews; no diagnosis rested on the MMPI alone, and we explicitly avoid any interpretation that would frame a transgender identity itself as psychopathology. Third, because scoring was not standardized, the profile categories cannot be benchmarked against normative or clinical comparison groups, and any apparent difference between subgroups should be read as hypothesis-generating only. For these reasons we report the MMPI results descriptively, avoid inferential claims about them, and would encourage future studies to use standardized, trans-inclusive instruments. We note that the concern is not that trans people necessarily differ psychometrically, but that several MMPI items and validity indicators are keyed to binary sex-assigned-at-birth norms, so scale elevations can reflect the instrument’s assumptions rather than clinical status; trans-inclusive validation or norms would allow raw scores to be used without this confounding. 

Geographic access showed an association that could easily reflect unmeasured confounders—socioeconomic status chief among them, since we had no income or education data to check against it. Province of residence is at best a crude proxy for the material barriers that likely drive the effect (travel time, lost wages, accommodation, and the capacity to attend repeated appointments), and these are patterned by income and employment in ways we could not model; the association is therefore correlational, not causal, and the specific mechanism remains untested. Follow-up continuity in trans populations is known to be fragile even in better-resourced settings [45], which makes it plausible that distance compounds pre-existing access disadvantages rather than acting alone. Follow-up data more broadly were anchored to the point of presentation rather than tracked forward, so nothing here speaks to long-term postsurgical outcomes. 

One further gap is a matter of timing rather than method: the data window closed at the end of 2024, just before regulatory changes reported for Türkiye in 2024/2025 (including a higher minimum-age threshold for hormone therapy and greater centralization of board authority). Their impact on care trajectories will fall to future cohorts to document. 

Several further limitations should also be noted. Outcomes completed outside the center—particularly legal name change—may be under-ascertained, so stage attainment could be underestimated. The care stages are not strictly nested: twelve individuals obtained a legal name change without surgical-board evaluation and two reached the surgical board without a hormone-board record, so the pathway is better described as partly parallel than as a strict cascade. The Kaplan–Meier analyses assume non-informative censoring, which may not hold if patients who stopped attending or transferred care differ from those still in follow-up. Province of residence is an ecological proxy that cannot separate geographic access from referral-source, socioeconomic, or health-system differences. Because patient goals and preferences were not documented, non-attainment of a stage should not be equated with dropout or loss from care—some individuals may not have sought that stage, may have completed it elsewhere, or may still have been waiting at the cutoff. Clinic capacity, staffing, documentation systems, and legal practice may all have changed across the 2010–2024 window, and the COVID-19 pandemic overlapped the later years and may have affected presentations and service availability. Finally, nine participants were younger than 18 at presentation, so describing the sample as strictly adult is an approximation; an adult-only sensitivity analysis (*n* = 188) left all main findings essentially unchanged. 

Additional limitations concern unavailable or incompletely recorded outcomes. First, we had no systematic data on regret or on discontinuation of transition-related interventions; these outcomes were not recorded in a retrievable form, so we cannot describe longer-term clinical trajectories. Second, individual surgical procedures could not be reliably tracked: many are performed at other centers or were not systematically recorded in the clinical file, which is why board referral rather than completed surgery was used as the outcome. Third, sex assigned at birth was recorded as a binary field and intersex status was not separately elicited, so any intersex individuals could not be identified. Fourth, non-binary identities could not be distinguished in the records, as the clinical fields encoded a binary framework; the resulting inability to identify non-binary people is a limitation that also constrains the affirming scope of the analysis. 

## 5. Conclusions

Referrals for gender dysphoria increased overall across this 15-year period at a university-based center in Türkiye, although the rise was driven largely by the early years and flattened after 2017. The trends reported in the Western literature—younger age at referral over time and a growing share of trans men—did not appear here. The largest decrease along the pathway occurred at the first step, and residence outside the center’s province was associated with lower attainment of surgical-board evaluation in exploratory analysis, an association that did not persist in time-to-event modeling. 

Western cohort data, then, cannot simply be transplanted onto a setting with a different legal and cultural landscape. What is needed instead is evidence built from adult, non-Western populations themselves, paired with further evaluation of the geographic and structural barriers to specialized care faced by patients living outside the center’s province. 

### Implications for Practice and Policy

The pathway data point to three places where practice and policy might reasonably be examined. The largest decrease occurred at intake, which suggests that psychosocial support and referral processes at the start of the pathway may warrant particular attention, since measures concentrated at later stages would not address the stage where most non-progression is observed. This inference rests on where the decrease occurs rather than on any direct test of an intervention and would require prospective evaluation. 

Residence was also associated with attainment in exploratory analysis. Individuals living outside the center’s province showed lower surgical-board attainment under logistic regression, which—if confirmed—might support evaluating telepsychiatry and hybrid follow-up models, which have been proposed for improving access in transgender care [46]. Reimbursement policy could similarly be examined: coverage currently stops short of indirect costs such as transportation and accommodation, which, while not measured here, could plausibly bear on whether a distant patient can attend follow-up. These are possible responses requiring evaluation, not interventions demonstrated by the present study. 

A third consideration is the structural requirement built into mandatory psychiatric and forensic steps. The prior literature reports that such requirements can be experienced as slow and, at times, alienating, and describes how mandatory psychological evaluation shapes patient experience [47]; the present study did not collect patient-reported experience and cannot speak to this directly. Closer alignment with international standards such as SOC-8 might help address this; international policy environments for gender-affirming care have themselves fluctuated [48], underscoring that any such alignment would require formal evaluation. 

## Figures and Tables

**Figure 1:**
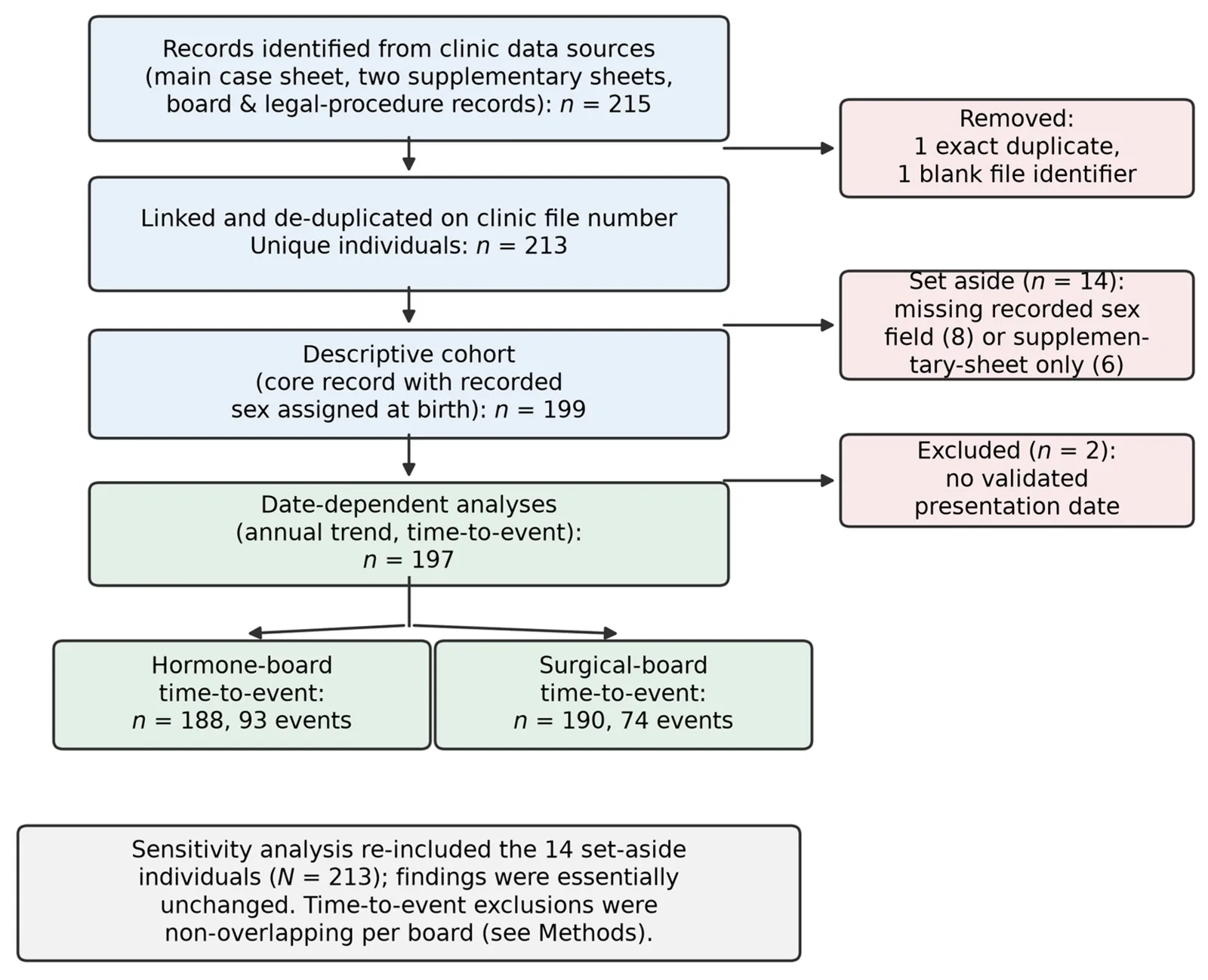
Study flow diagram (STROBE): from record screening to the main analytic cohort (*N* = 199) and the sensitivity-analysis cohort (*N* = 213).

**Figure 2:**
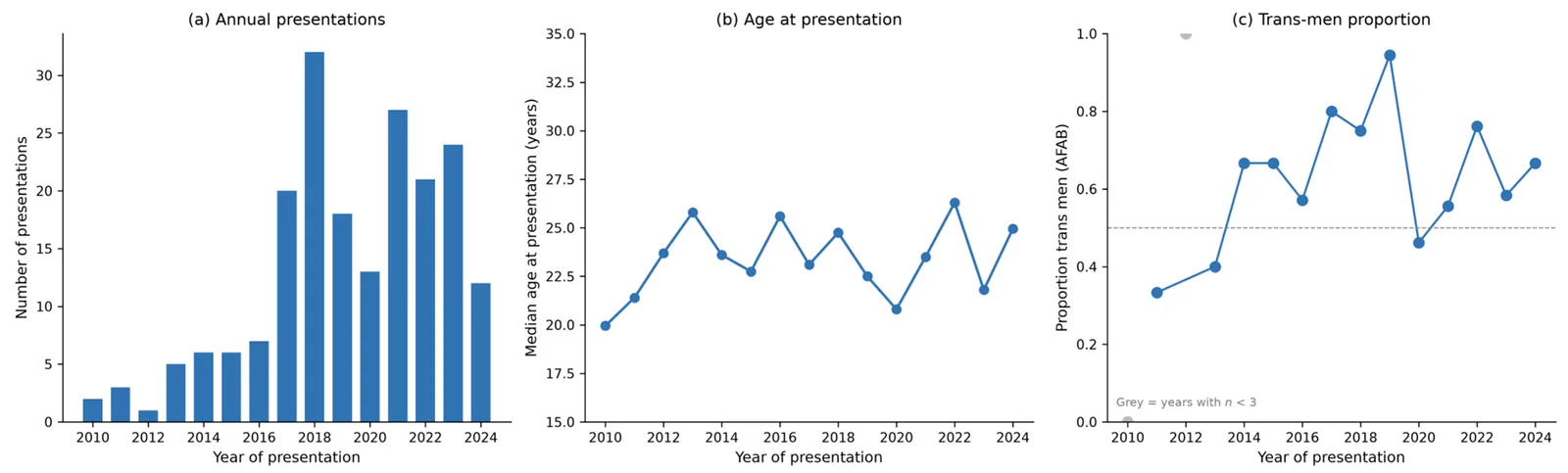
Presentation trends, 2010–2024. (**a**) Annual number of presentations by sex assigned at birth; (**b**) age at presentation remaining stable over time; and (**c**) proportion of trans men remaining predominant but not changing significantly over time (95% Wilson confidence intervals; all years are shown, with years having *n* < 3 marked in gray).

**Figure 3:**
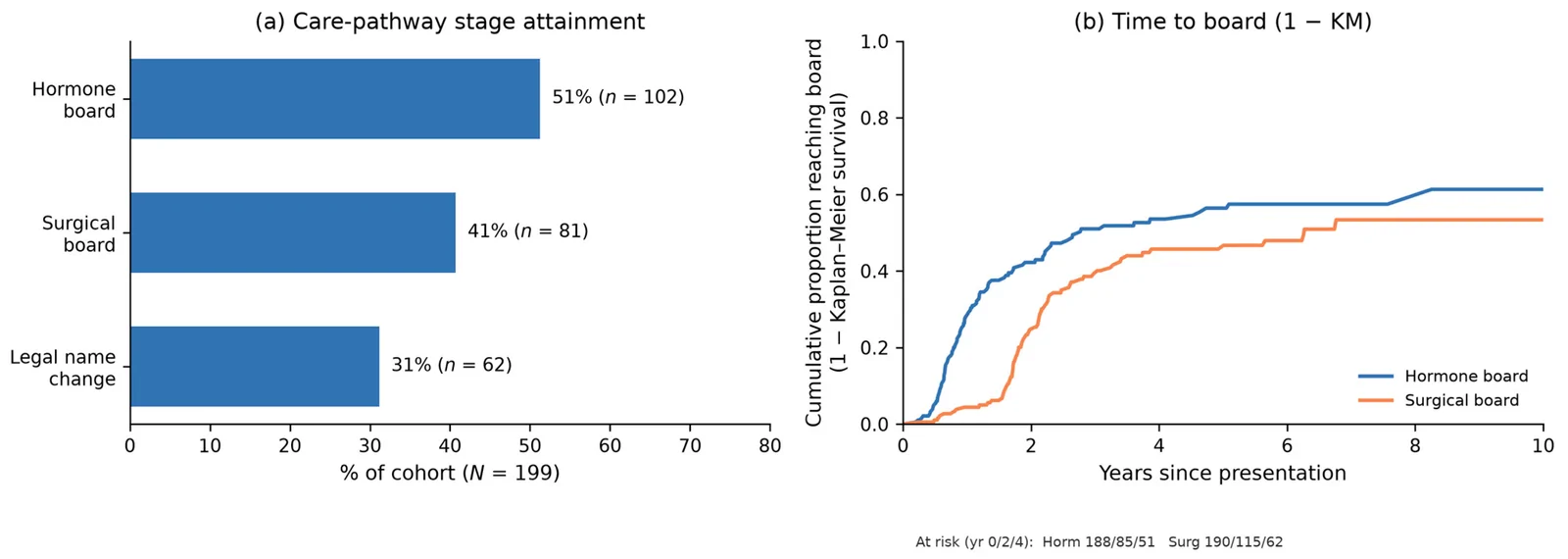
Gender-affirming care pathway. (**a**) Number of individuals reaching each stage (horizontal bar chart, of *N* = 199); (**b**) time from presentation to reaching each board, shown as cumulative incidence (1—Kaplan–Meier survival) with numbers at risk annotated.

**Figure 4:**
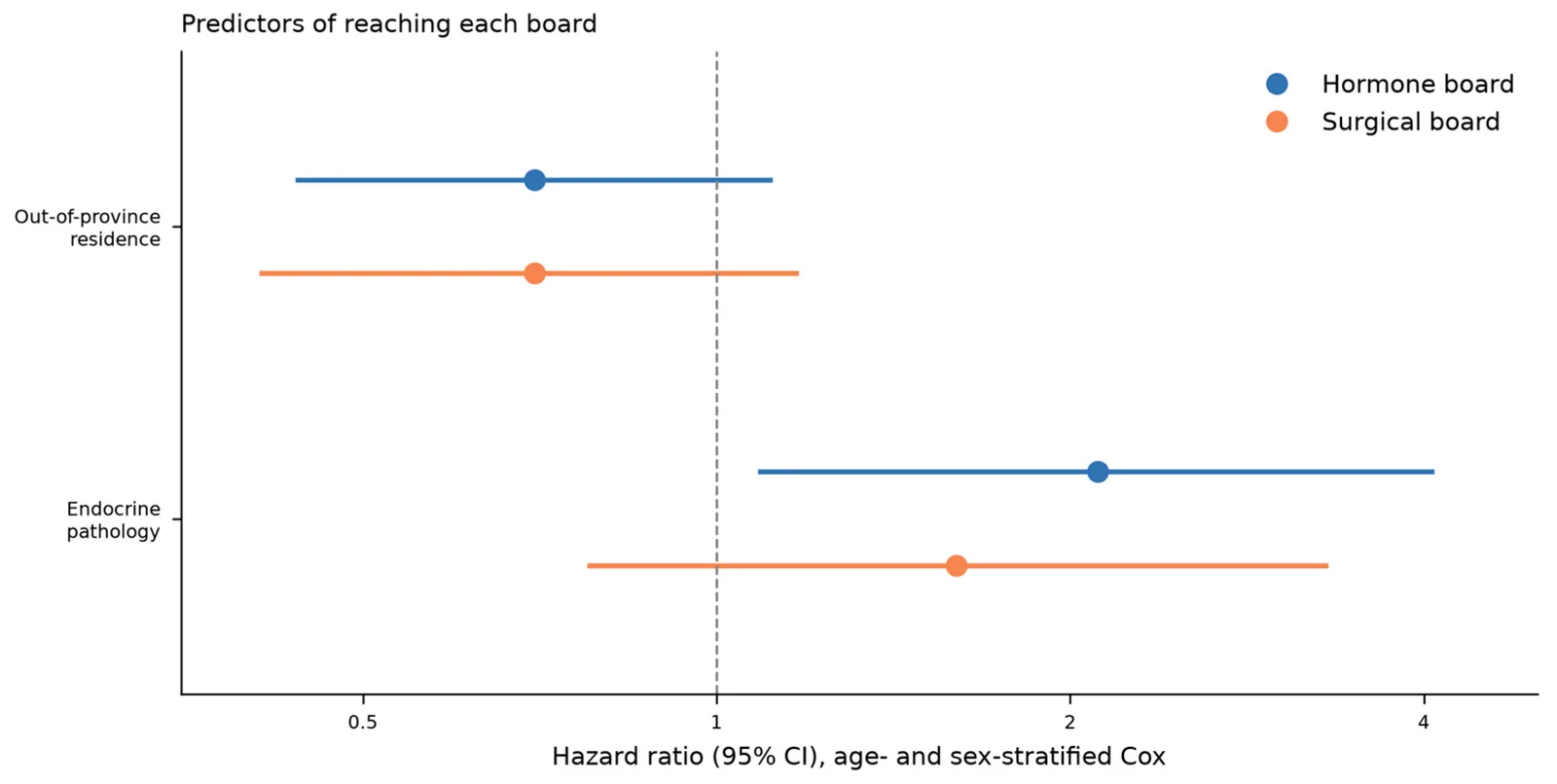
Predictors of reaching each board, shown as hazard ratios (95% CI) from Cox proportional-hazards models. Values to the right of 1 indicate faster progression.

**Figure 5:**
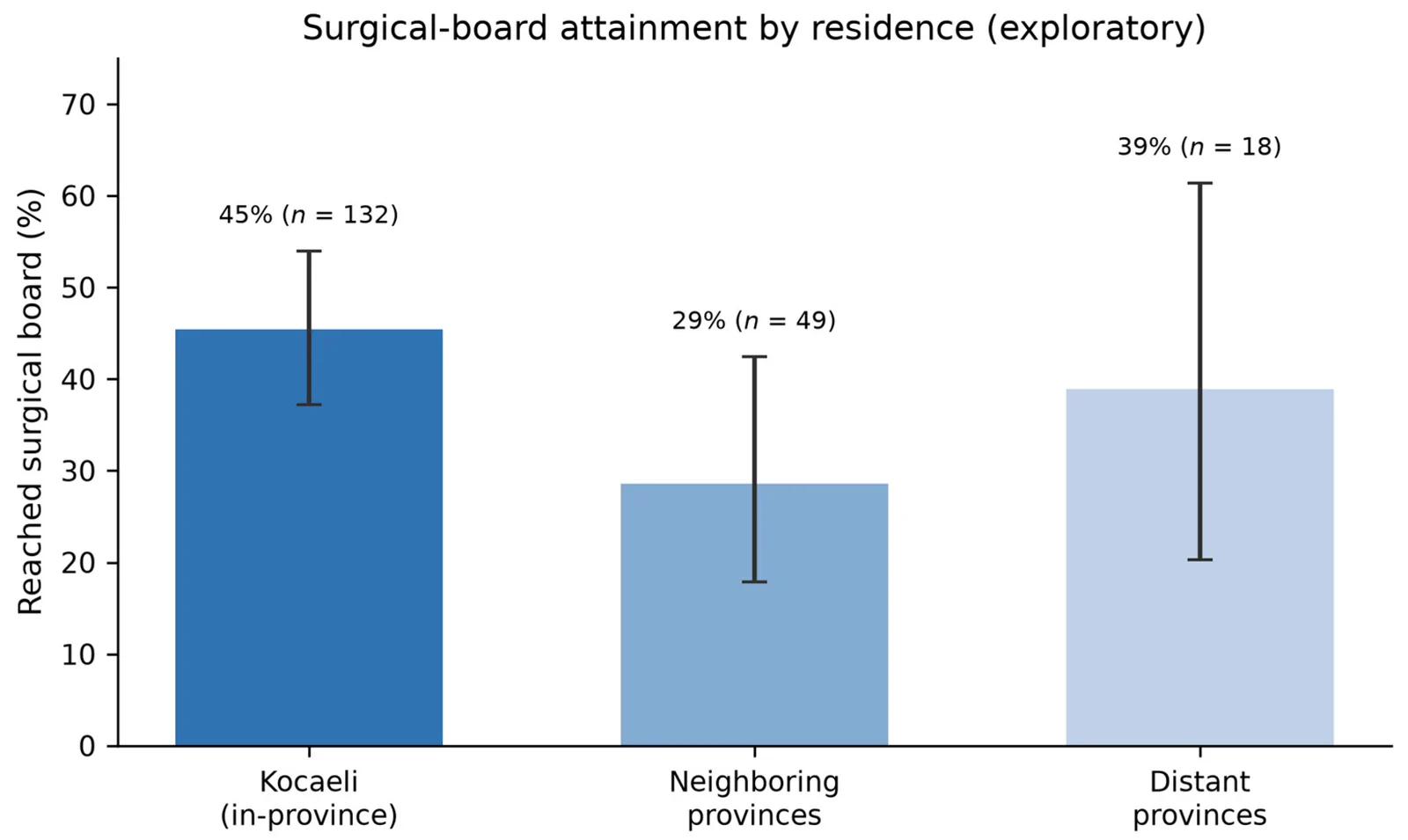
Surgical-board attainment by geographic tier (exploratory; 95% Wilson confidence intervals; *n* per tier shown).

**Table 1:** Sociodemographic and clinical characteristics of the cohort, overall and by sex assigned at birth (*N* = 199).

Variable	Total	Trans Men (AFAB)	Trans Women (AMAB)	Effect Size	Difference (Trans Men—Trans Women), 95% CI	*p*	Analytic *N* (Missing)
Age at presentation, median (IQR)	23.3 (20.0–27.6)	23.6 (20.8–27.6)	22.5 (18.8–27.5)	r = +0.13	+1.1 (−0.9–+3.4)	0.154	197 (2)
Age at diagnosis, median (IQR)	23.0 (19.0–27.0)	23.0 (20.0–27.0)	22.0 (18.0–27.0)	r = +0.12	+1.0 (−1.0–+3.0)	0.179	197 (2)
Residence in Kocaeli, *n* (%)	132 (66.3%)	83 (61.9%)	49 (75.4%)	OR 0.54	(0.28–1.04)	0.085	199 (0)
Endocrine pathology, *n* (%)	14 (7.0%)	12 (9.0%)	2 (3.1%)	OR 2.59	(0.64–10.42)	0.221	199 (0)
Reached hormone board, *n* (%)	102 (51.3%)	71 (53.0%)	31 (47.7%)	OR 1.23	(0.68–2.22)	0.583	199 (0)
Reached surgical board, *n* (%)	81 (40.7%)	58 (43.3%)	23 (35.4%)	OR 1.38	(0.75–2.54)	0.363	199 (0)
Legal name change, *n* (%)	62 (31.2%)	46 (34.3%)	16 (24.6%)	OR 1.58	(0.81–3.05)	0.221	199 (0)
Group therapy, *n* (%)	94 (47.2%)	69 (51.5%)	25 (38.5%)	OR 1.69	(0.93–3.07)	0.115	199 (0)

AFAB, assigned female at birth; AMAB, assigned male at birth; IQR, interquartile range; OR, odds ratio; and CI, confidence interval.

**Table 2:** Referral trends (2010–2024): Statistical tests and effect sizes.

Analysis	Method	Estimate (95% CI)	*p*	Note
Presentations/year (overall)	Negative binomial	IRR 1.21 (1.06–1.37)	0.003	Overall increase; overdispersed (χ^2^/df = 4.5)
Presentations/year (overall)	Poisson (for comparison)	IRR 1.14 (1.10–1.18)	<0.001	Underestimates uncertainty
Slope from 2015	Negative binomial	IRR 1.07 (0.86–1.34)	0.54	Weakened
Slope from 2017	Negative binomial	IRR 0.96 (0.70–1.31)	0.78	Flat/plateau
Pre/post-2017 mean count	Descriptive	4.3 → 20.9 per year	—	Descriptive only, not causal
Age at presentation trend	Linear/Spearman	ρ ≈ 0 (Spearman)	0.997	No change
Proportion trans-men trend	Logistic regression	OR 1.02/year (0.93–1.12)	0.674	No change

IRR, incidence rate ratio; CI, confidence interval.

**Table 3:** Cross-tabulation of care-pathway stage combinations (*N* = 199).

Hormone Board	Surgery Board	Legal Name Change	*n*	% of 199
No	No	No	85	42.7
Yes	Yes	Yes	50	25.1
Yes	Yes	No	29	14.6
Yes	No	No	21	10.6
No	No	Yes	10	5.0
Yes	No	Yes	2	1.0
No	Yes	No	2	1.0

**Table 4:** Predictors of reaching each board (Cox proportional-hazards models, both stratified by age group and sex assigned at birth, whose effects were time-varying).

Outcome	Predictor	HR	95% CI	*p*
Hormone board	Out-of-province residence	0.70	0.44–1.11	0.128
Hormone board	Endocrine pathology	2.11	1.09–4.06	0.026
Surgery board	Out-of-province residence	0.70	0.41–1.17	0.174
Surgery board	Endocrine pathology	1.60	0.78–3.30	0.201

Both models were stratified by age group (<20, 20–25, and >25 years) and sex assigned at birth. Hormone board: 188 individuals, 93 events; surgical board: 190 individuals, 74 events. The endocrine-pathology estimate was confirmed with penalized (ridge) Cox regression (hormone board penalized HR 1.93, *p* = 0.042; surgical board penalized HR 1.51, *p* = 0.24). Fourteen individuals reaching a board without a recorded date were excluded as events with unknown timing. HR, hazard ratio; CI, confidence interval.

## Data Availability

The datasets generated and analyzed during the current study are not publicly available because they contain sensitive clinical information on a small and potentially identifiable population of transgender individuals, and public release could compromise participant privacy. De-identified data are available from the corresponding author on reasonable request, subject to approval by the Kocaeli University Non-Interventional Clinical Research Ethics Committee and compliance with applicable data-protection regulations.

## Supplementary Materials

The following supporting information can be downloaded at: https://www.mdpi.com/article/10.3390/healthcare14193144/s1, Figure S1: Distribution of clinician-interpreted MMPI profiles (*n* = 143 assessed). Testing was selective and interpretations were non-standardized; presented descriptively; Table S1: Clinician-interpreted MMPI profiles among those assessed (*n* = 143); Table S2: Multivariable logistic-regression models (odds ratios) for reaching each stage, adjusted for follow-up duration, reported for comparability with prior work; the Cox models in the main text are primary; Table S3: Annual number of first presentations, 2010–2024 (used for the trend analysis).

## Abbreviations

The following abbreviations are used in this manuscript:

AFAB	Assigned female at birth
AMAB	Assigned male at birth
CI	Confidence interval
ENIGI	European Network for the Investigation of Gender Incongruence
IQR	Interquartile range
IRR	Incidence rate ratio
MMPI	Minnesota Multiphasic Personality Inventory
OR	Odds ratio
SOC-8	Standards of Care, Version 8
WPATH	World Professional Association for Transgender Health
