# Supplementary material for: Fifteen-Year Referral Trends and the Gender-Affirming Care Pathway in a Predominantly Adult Gender Dysphoria Cohort: A Retrospective Cohort Study from a University-Based Center in Türkiye
Source: Healthcare (Basel). 2026 Sep 22;14(19):3144. doi: 10.3390/healthcare14193144 (PMC13664777; doi:10.3390/healthcare14193144)
Supplement: Supplementary file 1 [file healthcare-14-03144-s001.zip › healthcare-4507512-supplementary.pdf]

Supplementary Materials

Fifteen-Year Referral Trends and the Gender-Affirming Care Pathway in a Predominantly Adult Gender Dysphoria Cohort: A Retrospective Cohort Study from a University-Based Center in Türkiye

Figure S1. Distribution of clinician-interpreted MMPI profiles (*n* = 143 assessed). Testing was selective and interpretations were non-standardized; presented descriptively.

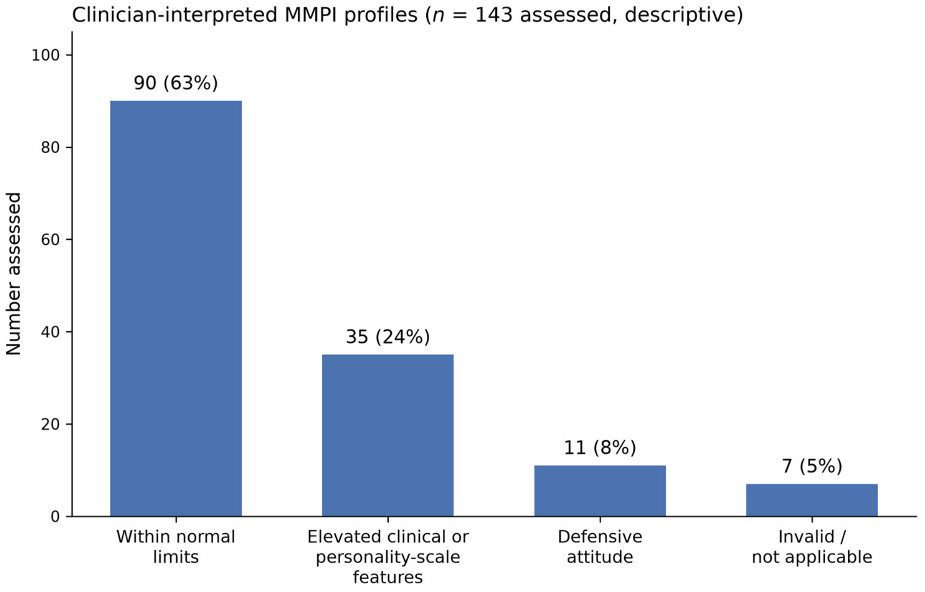

Table S1. Clinician-interpreted MMPI profiles among those assessed (*n* = 143).

Main category	Subtype	n	% (of those tested, *n*=143)
Within normal limits		90	62.9
Elevated clinical or personality-scale features (total)		35	24.5
	— Personality features (Cluster B / borderline / antisocial / histrionic / passive / narcissistic / avoidant)	18	12.6
	— Neurotic profile	12	8.4
	— Other elevated subscale (anger, low frustration tolerance)	3	2.1
	— Mood/anxiety/somatic	2	1.4
Defensive attitude		11	7.7
Invalid / not applicable		7	4.9

Table S2. Multivariable logistic-regression models (odds ratios) for reaching each stage, adjusted for follow-up duration, reported for comparability with prior work; the Cox models in the main text are primary.

**Outcome**	**Predictor**	**OR**	**95% CI**	***p***
Hormone board	Trans man (vs trans woman)	1.29	0.67–2.45	0.444
Hormone board	Out-of-province residence	0.59	0.31–1.12	0.107
Hormone board	Age at presentation (per year)	1.02	0.97–1.06	0.510
Hormone board	Endocrine pathology	6.79	1.41–32.73	0.017
Hormone board	Follow-up duration (per year)	1.2	1.09–1.33	<0.001
Surgery board	Trans man (vs trans woman)	1.6	0.81–3.16	0.178
Surgery board	Out-of-province residence	0.5	0.25–0.98	0.045
Surgery board	Age at presentation (per year)	1.02	0.97–1.06	0.475
Surgery board	Endocrine pathology	3.05	0.90–10.36	0.074
Surgery board	Follow-up duration (per year)	1.25	1.13–1.39	<0.001
Legal name change	Trans man (vs trans woman)	1.68	0.82–3.44	0.155
Legal name change	Out-of-province residence	0.79	0.40–1.57	0.509
Legal name change	Age at presentation (per year)	1.03	0.98–1.08	0.200
Legal name change	Endocrine pathology	1.62	0.50–5.21	0.422
Legal name change	Follow-up duration (per year)	1.18	1.07–1.31	0.001

Table S3. Annual number of first presentations, 2010–2024 (used for the trend analysis).

Year	First presentations (*n*)
2010	2
2011	3
2012	1
2013	5
2014	6
2015	6
2016	7
2017	20
2018	32
2019	18
2020	13
2021	27
2022	21
2023	24
2024	12
**Total**	**197**

STROBE Statement — Checklist for Cohort Studies

Manuscript: Fifteen-Year Referral Trends and the Gender-Affirming Care Pathway in a Predominantly Adult Gender Dysphoria Cohort: A Retrospective Cohort Study from a University-Based Center in Türkiye. Locations are given by manuscript section in the line-numbered submission.

Section / Item	No.	Recommendation	Reported in manuscript (section)
Title and abstract	1(a)	Study design indicated in title/abstract	Title (retrospective cohort study); Abstract
Title and abstract	1(b)	Balanced informative abstract	Structured abstract (Background/Objectives, Methods, Results, Conclusions)
Introduction — Background/rationale	2	Scientific background and rationale	Introduction §1
Introduction — Objectives	3	Specific objectives / hypotheses	Introduction §1 (final paragraph)
Methods — Study design	4	Key design elements early in paper	Methods §2.1
Methods — Setting	5	Setting, locations, relevant dates (recruitment, exposure, follow-up, data collection)	Methods §2.1 (2010–2024; database closing 25 Nov 2024)
Methods — Participants	6(a)	Eligibility criteria; sources and selection methods	Methods §2.1 (the descriptive cohort required a core clinical record with recorded sex assigned at birth; a validated presentation date was required only for date-dependent analyses); Figure 1.
Methods — Participants	6(b)	(Matched studies) matching criteria — not applicable	Not applicable (not a matched design)
Methods — Variables	7	Outcomes, exposures, predictors, confounders, effect modifiers; diagnostic criteria	Methods §2.2 (operational definitions: care-pathway stages, geographic tiers, endocrine pathology, age at diagnosis)
Methods — Data sources/measurement	8	Sources of data and measurement methods for each variable	Methods §2.1–2.2 (chart abstraction from case spreadsheet, supplementary sheets, board and clinical records)
Methods — Bias	9	Efforts to address potential sources of bias	Methods §2.3 (a priori adjustment set; indication-bias disclosure for MMPI); Limitations §4.1
Methods — Study size	10	How study size was arrived at	Methods §2.1 (all eligible individuals 2010–2024); Figure 1
Methods — Quantitative variables	11	Handling of quantitative variables; groupings and why	Methods §2.2–2.3 (age stratified <20/20–25/>25; geographic tiers)
Methods — Statistical methods	12(a)	All methods, including confounding control	Methods §2.3 (negative-binomial; Cox stratified by age and sex; a priori covariates)

Section / Item	No.	Recommendation	Reported in manuscript (section)
Methods — Statistical methods	12(b)	Methods for subgroups and interactions	Methods §2.3 (sub-period trend slopes; sex-stratified models)
Methods — Statistical methods	12(c)	How missing data were addressed	Methods §2.3 (complete-case); Table 1 (per-variable analytic N)
Methods — Statistical methods	12(d)	How loss to follow-up / censoring addressed	Methods §2.3 (administrative censoring at 25 Nov 2024; non-informative-censoring assumption; known events without dates excluded)
Methods — Statistical methods	12(e)	Sensitivity analyses	Methods §2.3; Results §3.7 (N=213; adult-only n=188; penalized Cox; exclusion of undated events)
Results — Participants	13(a)	Numbers at each stage (eligible, examined, analyzed)	Results §3.1; Figure 1 (215→213→199)
Results — Participants	13(b)	Reasons for non-participation	Figure 1; Results §3.1 (one duplicate and one blank-identifier record removed; 14 individuals set aside: 8 with missing recorded sex and 6 with supplementary-sheet-only records; 2 lacked a validated presentation date and were excluded only from date-dependent analyses).
Results — Participants	13(c)	Flow diagram	Figure 1 (STROBE flow)
Results — Descriptive data	14(a)	Characteristics, exposures, confounders	Table 1; Results §3.1
Results — Descriptive data	14(b)	Missing data per variable	Table 1 (analytic N / missing column)
Results — Descriptive data	14(c)	Follow-up time (summary)	Results §3.3: median follow-up 4.9 years (IQR 2.5–6.9; range 0.0–14.5; 1008 person-years); crude vs Kaplan-Meier medians and numbers at risk reported
Results — Outcome data	15	Numbers of outcome events or summary measures over time	Results §3.3; Table 3 (cross-tabulation); Figure 3
Results — Main results	16(a)	Unadjusted and adjusted estimates with CIs and confounders adjusted for	Results §3.4; Table 4 (Cox HR); Supplement Table S2 (logistic OR)
Results — Main results	16(b)	Category boundaries when continuous variables categorized	Methods §2.2 (age and geographic-tier boundaries)
Results — Main results	16(c)	Absolute risk translation if relevant	Results §3.3 (attainment percentages; not further translated)

Section / Item	No.	Recommendation	Reported in manuscript (section)
Results — Other analyses	17	Subgroups, interactions, sensitivity analyses	Results §3.5 (geography, exploratory); §3.7 (sensitivity); Supplement (MMPI, indication bias)
Discussion — Key results	18	Summarize key results with reference to objectives	Discussion §4 (opening)
Discussion — Limitations	19	Limitations; direction and magnitude of bias	Limitations §4.1
Discussion — Interpretation	20	Cautious overall interpretation	Discussion §4; §4.1
Discussion — Generalizability	21	External validity	Discussion §4; §4.1 (single-center, Türkiye, adult-service)
Other — Funding	22	Funding and role of funders	Back matter (Funding statement)
